# Advanced glycation end products are not associated with bone mineral density, trabecular bone score, and bone turnover markers in adults with and without type 1 diabetes: a cross-sectional study

**DOI:** 10.1093/jbmrpl/ziad018

**Published:** 2024-01-04

**Authors:** Julie-Catherine Coll, Anne-Frédérique Turcotte, William D Leslie, Laëtitia Michou, Stanley John Weisnagel, Fabrice Mac-Way, Caroline Albert, Claudie Berger, Suzanne N Morin, Rémi Rabasa-Lhoret, Claudia Gagnon

**Affiliations:** Centre de recherche, CHU de Québec-Université Laval, Quebec City, QC G1V 4G2, Canada; Centre de recherche, CHU de Québec-Université Laval, Quebec City, QC G1V 4G2, Canada; Department of Medicine, University of Manitoba, Winnipeg, MB R3T 2N2, Canada; Centre de recherche, CHU de Québec-Université Laval, Quebec City, QC G1V 4G2, Canada; Department of Medicine, Université Laval, Quebec City, QC G1V 0A6, Canada; Centre de recherche, CHU de Québec-Université Laval, Quebec City, QC G1V 4G2, Canada; Department of Medicine, Université Laval, Quebec City, QC G1V 0A6, Canada; Centre de recherche, CHU de Québec-Université Laval, Quebec City, QC G1V 4G2, Canada; Department of Medicine, Université Laval, Quebec City, QC G1V 0A6, Canada; Centre Hospitalier de l’Université de Montréal, Montreal, QC H2X 3E4, Canada; Research Institute of the McGill University Health Centre, Montreal, QC H4A 3J1, Canada; Research Institute of the McGill University Health Centre, Montreal, QC H4A 3J1, Canada; Department of Medicine, McGill University, Montreal, QC H4A 3J1, Canada; Institut de recherches cliniques de Montréal, Montreal, QC H2W 1R7, Canada; Centre de recherche, CHU de Québec-Université Laval, Quebec City, QC G1V 4G2, Canada; Department of Medicine, Université Laval, Quebec City, QC G1V 0A6, Canada

**Keywords:** AGEs, bone mineral density, trabecular bone score, biochemical markers of bone turnover, type 1 diabetes

## Abstract

It is unclear if AGEs are involved in the bone fragility of type 1 diabetes (T1D). We evaluated whether skin AGEs by skin autofluorescence and serum AGEs (pentosidine, carboxymethyl-lysine [CML]) are independently associated with BMD by DXA (lumbar spine, hip, distal radius), trabecular bone score (TBS), serum bone turnover markers (BTMs: CTX; P1NP; osteocalcin), and sclerostin in participants with and without T1D. Linear regression models were used, with interaction terms to test effect modification by T1D status. In participants with T1D, correlations between skin and serum AGEs as well as between AGEs and 3-year HbA_1C_ were evaluated using Spearman’s correlations. Data are mean ± SD or median (interquartile range). We included individuals who participated in a cross-sectional study and had BMD and TBS assessment (106 T1D/65 controls, 53.2% women, age 43 ± 15 yr, BMI 26.6 ± 5.5 kg/m^2^). Participants with T1D had diabetes for 27.6 ± 12.3 yr, a mean 3-yr HbA_1C_ of 7.5 ± 0.9% and skin AGEs of 2.15 ± 0.54 arbitrary units. A subgroup of 65 T1D/57 controls had BTMs and sclerostin measurements, and those with T1D also had serum pentosidine (16.8[8.2–32.0] ng/mL) and CML [48.0 ± 16.8] ng/mL) measured. Femoral neck BMD, TBS, and BTMs were lower, while sclerostin levels were similar in participants with T1D vs controls. T1D status did not modify the associations between AGEs and bone outcomes. Skin AGEs were significantly associated with total hip and femoral neck BMD, TBS, BTMs, and sclerostin before, but not after, adjustment for confounders. Serum AGEs were not associated with any bone outcome. There were no significant correlations between skin and serum AGEs or between AGEs and 3-yr HbA_1C_. In conclusion, skin and serum AGEs are not independently associated with BMD, TBS, BTMs, and sclerostin in participants with relatively well-controlled T1D and participants without diabetes.

## Graphical Abstract

**Graphical Abstract:**
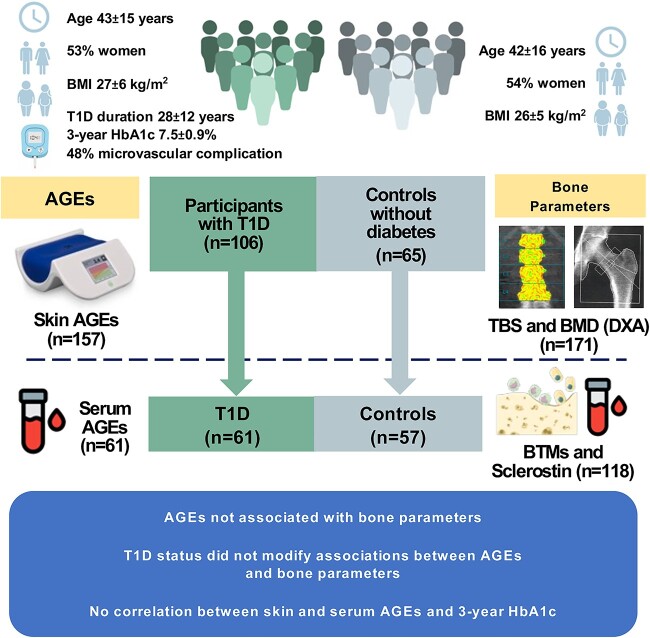

Skin AGEs and serum AGEs (pentosidine, carboxymethyl-lysine) were not associated with bone mineral density, trabecular bone score, serum bone turnover markers (CTX, P1NP, osteocalcin), and sclerostin in 106 participants with type 1 diabetes and 65 controls without diabetes. There was no correlation between skin and serum AGEs and mean 3-yr HbA1c.

## Introduction

Type 1 diabetes (T1D) significantly increases fracture risk at any site, with up to a 6-fold increase in hip fracture risk for individuals with T1D compared to those without diabetes.^1-5^ The mechanisms accounting for bone fragility in T1D are complex and still incompletely understood. Beyond reduced BMD,^6^ altered bone quality likely contributes to the high fracture rate observed in T1D via lower bone remodeling,^7^ altered bone microarchitecture,^8^ and abnormal material and biomechanical properties.^9^ A better understanding of the pathophysiology of reduced BMD and bone quality in T1D is needed to improve management.

AGEs are compounds produced by a nonenzymatic reaction between reducing sugars and the amine residues on proteins, lipids, and nucleic acids.^10^ AGEs are strongly involved in the pathogenesis of macrovascular and microvascular complications in T1D, and their tissue accumulation is accelerated by oxidative stress and hyperglycemia.^11^ Some of the best characterized AGEs in humans include pentosidine and N-carboxymethyl-lysine (CML). They accumulate in bone and after binding to the most abundant bone matrix protein, type 1 collagen, they cause cross-linked (pentosidine) and noncross-linked (CML) modifications of the collagen molecules, which alter the bone material properties.^12^ AGEs also inhibit osteoblast proliferation, induce osteoblast apoptosis, decrease osteoclast-induced bone resorption, as well as the osteoclastic differentiation process.^13^ Altogether, these data suggest that AGEs could play a role in the bone fragility of people with T1D. In support of this, it has been reported that trabecular bone from subjects with T1D with a prior fragility fracture exhibited higher pentosidine content than trabecular bone from nondiabetic controls without a history of fracture.^14^ Another study identified that higher skin AGEs were independently associated with a lower total hip, femoral neck, and ultra-distal radius areal BMD in T1D.^15^ Moreover, serum pentosidine levels were independently associated with prevalent fractures in men and premenopausal women with T1D.^16^ However, the exact mechanisms by which AGEs accumulation could alter bone quality and BMD and thus contribute to the higher fracture risk observed in T1D remain to be elucidated.

AGEs can be measured in serum, urine, and various tissues, among which skin is of particular interest. Indeed, skin AGEs exhibit intrinsic fluorescent properties that enable assessment by skin autofluorescence, a simple, quick, noninvasive, and highly reproducible method.^17^ Moreover, skin AGEs measured by autofluorescence have been correlated with both fluorescent (pentosidine) and nonfluorescent (CML) AGEs measured in skin biopsies,^18^^,^^19^ which, in turn, have been correlated to bone pentosidine levels in studies on human cadavers.^20^ Skin AGEs by skin autofluorescence could then represent a practical and noninvasive surrogate marker of bone AGEs. Finally, skin AGEs are a better predictor of T1D complications than glycated hemoglobin (HbA_1C_),^21-23^ which may be attributed to the fact that skin AGEs track long-term glycemic control.

Lumbar spine trabecular bone score (TBS) provides an indirect assessment of bone structure and fracture risk, independently of BMD.^24^ We previously reported in a cross-sectional study that total hip and femoral neck BMD, TBS, and bone turnover markers (BTMs) were lower in people with T1D compared with controls without diabetes with a similar age, sex, and BMI distribution, which suggest reduced BMD and bone quality could contribute to bone fragility in T1D.^25^ We now aim to determine whether skin AGEs, assessed by skin autofluorescence, are independently associated with BMD by DXA, TBS, BTMs, and sclerostin in people with T1D and controls, while evaluating if T1D status modifies these associations. As secondary objectives, we aim to evaluate in a subgroup of participants with T1D (1) if serum AGEs (pentosidine, CML) are independently associated with BMD by DXA, TBS, BTMs, and sclerostin; (2) if there is a correlation between skin and serum AGEs; and (3) if skin and serum AGEs correlate with mean HbA_1C_ over 3 yr.

## Materials and methods

### Study design and participants

This study stems from a multicenter, cross-sectional study aiming at comparing the prevalence of vertebral fractures between adults with T1D and controls without diabetes with a similar age, sex, and BMI distribution.^25^ The study was registered at https://clinicaltrials.gov/ct2/show/NCT04064437. It was approved by local ethics committees and conducted in accordance with the World Medical Association Declaration of Helsinki. All participants provided written informed consent.

Details of the methodology can be found in the original publication^25^ and are summarized here. The study was conducted at the CHU de Québec-Université Laval in Quebec City and at the Clinical Research Institute of Montreal (IRCM) in Montreal and included 127 participants with T1D and 65 controls. Only the participants evaluated in Quebec City (106 with T1D, 65 controls) had TBS assessment as well as BTMs and serum AGEs measurements; hence, only this sample contributed to the present study.

Participants with T1D were recruited in the outpatient endocrinology diabetes clinics from July 2019 to March 2021. They were included if aged ≥20 yr and had been diagnosed with T1D for at least 5 yr. They were excluded if they presented conditions or drugs affecting bone metabolism; however, participants with chronic kidney disease or celiac disease were included as these conditions are frequently encountered in people with T1D.

Controls without diabetes aged ≥20 yr were recruited among former participants from other research studies as well as among the CHU de Québec-Université Laval and Laval University employees and students. Exclusion criteria were the same as in participants with T1D plus: diagnosis of prediabetes (HbA_1C_ ≥ 6.0%) or diabetes as per the Diabetes Canada Clinical Practice Guidelines criteria,^26^ chronic kidney disease (estimated glomerular filtration rate [eGFR] <60 mL/min/1.73 m^2^), celiac disease, and past medical history of a low-trauma fracture.

### Clinical assessment

An interview was conducted to collect clinical data, and each participant completed self-administered questionnaires on socioeconomic status, osteoporosis risk factors, hypoglycemic events, supplements and medication use, and diabetes management and complications. Past medical history of fracture was also self-reported, and all past fractures, regardless of age, bone site, and mechanism, were collected. A fracture resulting from a fall from standing height or from a minimal trauma (eg, coughing, turning over) was defined as a low-trauma fracture. All data were validated in the patient electronic medical record, when possible. A mean HbA_1C_ over the 3 yr prior to the visit was calculated for participants with T1D with all available values from the electronic medical record. The presence of diabetes complications at the last outpatient visit with the endocrinologist was also ascertained in the patient electronic medical record. Diabetic retinopathy, nephropathy, and neuropathy were screened and defined as per the most recent Diabetes Canada Clinical Practice Guidelines.^26^ A physical examination, which included anthropometric measurements (height, weight, and waist circumference^27^) and a peripheral neuropathy screening with monofilament and vibration testing,^28^ was performed.

### BTMs and biochemistry

BTMs and sclerostin were measured among a subgroup of participants with T1D (*n* = 61) and controls (*n* = 57) who had their blood sampled in the morning before 10:00 am after a minimum 10-h fast. Serum samples were frozen at −80 °C until batch analysis at the study end. Serum CTX [CV <4%], P1NP [CV <2%], and N-MID osteocalcin [CV <2%] were measured by automated electrochemiluminescence immunoassay (Cobas e411, Roche Diagnostics). These measurements were performed at the Centre Hospitalier de l’Université de Montréal, a clinical laboratory with extensive experience in the measurement of BTMs. Serum sclerostin [CV 5%] was measured by ELISA using TECO kit, as per manufacturer’s protocol (Sclerostin TECO High Sensitive; TECOmedical Group).

All participants had PTH (Siemens Immulite 1000, Siemens Healthineers), 25OHD (Siemens Advia Centaur XPT, Siemens Healthineers; interassay CV 8%), and IGF-1 (Siemens Immulite 2000 xPi, Siemens Healthineers) measured by automated chemiluminescent immunoassay. Other biochemical tests were performed using standard automated techniques: HbA_1C_, hemoglobin, creatinine, lipid profile, thyrotropin (TSH), serum total calcium, albumin, phosphate, anti-transglutaminase antibodies and immunoglobulin A, and spot urine albumin-to-creatinine ratio. eGFR was calculated using the Chronic Kidney Disease Epidemiologic Collaboration equation.^29^

### Bone densitometry and TBS

DXA scans of the lumbar spine (L1–L4), total hip, femoral neck, and one-third distal radius were performed by trained technicians using a Hologic Discovery A densitometer (software version 13.5.1.3, Hologic Inc.). Quality control of the densitometer was performed using the manufacturer spine phantom each day participants were evaluated or minimally once a month. Sex-matched reference populations were used to calculate BMD T-scores and Z-scores (the National Health and Nutrition Examination Survey [NHANES] III^30^ for the femoral neck and total hip and the Hologic reference data for the lumbar spine and radius).

TBS provides an indirect index of trabecular architecture and is based on the extraction of a gray-level textural metric from the lumbar spine (L1–L4) DXA image.^31^ TBS iNsight Software version 3.0.3.0 (Medimaps Group) was used to compute TBS. TBS was calculated as the mean value of the individual vertebrae measurements from L1 to L4 and is unitless. TBS T-scores and Z-scores were calculated from sex-, age-, and ethnicity-matched reference values computed using a combined NHANES and Medimaps dataset.

### Skin and serum AGEs

Skin AGEs were measured by autofluorescence using the AGE Reader (Diagnoptics Technologies B.V.). It is a simple, noninvasive, rapid, and accurate technique during which the device illuminates a small area (~4 cm^2^) of the subject lower forearm skin.^18^ Ultraviolet light is emitted with a peak wavelength at 370 nm, exciting the AGEs in skin that have autofluorescence properties. The AGE Reader spectrophotometer measures the emission light with a wavelength of 420–600 nm and the reflected excitation light with a wavelength of 300–420 nm. Skin AGEs are expressed in arbitrary units (AU) as the ratio between the emission light intensity and the excitation light intensity multiplied by 100.^18^ Two consecutive measurements were performed on the underside of the right forearm to derive a mean value for each participant. If the participant had a tattoo or visible abnormalities (eg, scars, injuries) on the underside of the right forearm, the underside of the left forearm was used. When the skin reflectance percentage is <6%, which is the case of Fitzpatrick skin color classes V–VI (darker-skinned individuals), the measurement might be unreliable and then no measurement is given by the AGE Reader.^32^ All participants recruited had Fitzpatrick class I–IV skin color.

Serum pentosidine and CML were measured in the subgroup of participants with T1D (*n =* 61) who had their blood sampled in the morning after a minimum 10-h fast. Serum pentosidine was measured by ELISA using a commercial kit as per manufacturer’s protocol (Human Pentodisine ELISA Kit, code #ABX253063-96TESTS; Abbexa Ltd; intraassay and interassay CV <10%; sensitivity 0.47 ng/mL). Serum CML was measured by ELISA using a commercial kit as per manufacturer’s protocol (OxiSelect Nε-(carboxymethyl) lysine (CML) Competitive ELISA Kit, code #STA-816; Cell Biolabs Inc.; intraassay CV 4% and interassay CV 8%; sensitivity 2.25 ng/mL).

### Statistical analyses

Data were expressed as frequencies and percentages for categorical variables and as means ± SD or medians (interquartile range) for continuous variables, as appropriate. Characteristics between participants with T1D and controls were compared using unpaired *t*-tests or Mann–Whitney U tests for continuous variables and chi-squared tests or Fisher exact tests for categorical variables. Correlations were tested using unadjusted Spearman’s correlations and partial Spearman’s correlations adjusted for age and sex.

Simple linear regression analyses were performed as a first step to assess the associations between skin or serum AGEs and each bone parameter (BMD at the lumbar spine, total hip, femoral neck, and one-third distal radius, TBS, CTX, P1NP, osteocalcin, and sclerostin). Then, multiple linear regression analyses were conducted to adjust for potential confounders based on the literature. Directed acyclic graphs were designed to preclude the inclusion of intermediate variables in the models. Due to the expected collinearity between the BMI and waist circumference, and to the fact that tissue thickness is one of the strongest variables associated with significant TBS changes,^33^ the parameter between BMI and waist circumference that more strongly explained the TBS variance (with the highest *R*-squared value) in simple linear regression analysis was selected for the final adjusted model. To evaluate whether T1D status modifies the associations between AGEs and bone parameters, the interaction between T1D status and skin or serum AGEs was tested. In case of a nonsignificant interaction, participants with T1D and controls were pooled in the multiple linear regression analyses.

For the association between AGEs and BMD by DXA, the following confounders were successively added to the unadjusted models: age and menopausal status, BMI, T1D status, eGFR, and the presence of a microvascular complication. For the association between AGEs and TBS, the following confounders were successively added to the unadjusted model: age and sex, T1D status, waist circumference, eGFR, and presence of a microvascular complication. Waist circumference was preferred to BMI in the final model with TBS, as it better explained the TBS variance in simple linear regression analysis. Active smoking was not added to the models between AGEs and BMD or TBS, as it was not significantly associated with BMD at any site or TBS in simple linear regression analysis. For the association between AGEs and BTMs or sclerostin, the following confounders were successively added to the unadjusted models: age and menopausal status, T1D status, and eGFR.

To test the hypothesis that skin or serum AGEs might be associated with bone parameters in the situation of uncontrolled T1D, a sensitivity analysis was performed including the participants with T1D with an HbA_1C_ ≥ 7.8%. A 2-sided *P*-value <.05 was considered statistically significant. Statistical analyses were performed using the SAS software (version 9.4).

## Results

### Participants’ characteristics

#### Whole study population

**Figure 1:**
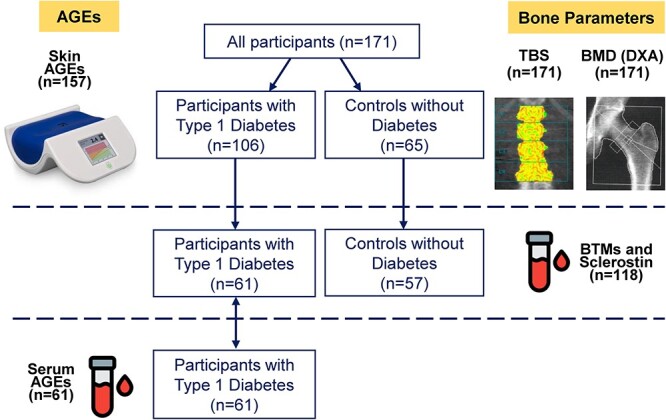
Flowchart of the bone parameters assessment and advanced glycation end products measurements among participants with and without type 1 diabetes. Figure Legend: All participants (*n =* 171) had BMD assessed by DXA and TBS and 157 participants had skin AGEs measurements. A subgroup of participants with type 1 diabetes and controls had serum BTMs and sclerostin measurements (*n =* 118). A subgroup of participants with type 1 diabetes had serum AGEs measurements (*n =* 61).

**Figure 2:**
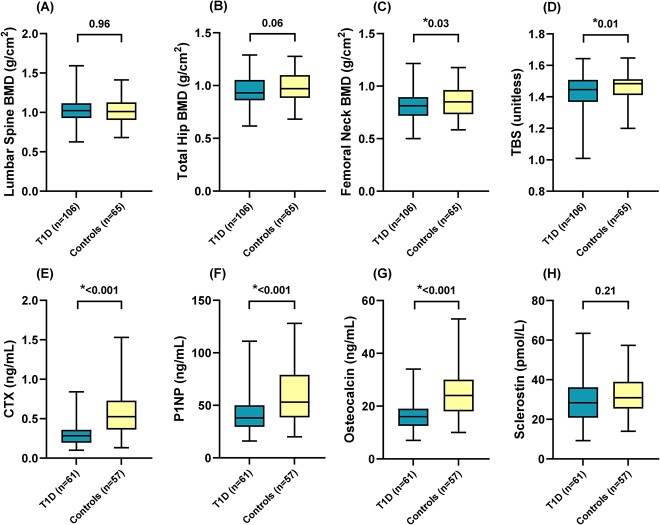
Bone parameters of participants with and without type 1 diabetes (*n =* 171). Figure Legend: Box and whisker plots (median, first, and third quartiles, minimum and maximum) are used to represent selected bone parameters in each group. *P*-values are from unpaired *t*-tests or Mann–Whitney U tests comparing means or medians between participants with type 1 diabetes and controls without diabetes. Significant *P*-values (*P* < .05) are marked with an asterisk (*).

A total of 171 participants (106 with T1D, 65 controls) were included. All participants had BMD and TBS assessment, a subgroup of participants with T1D and controls had BTMs and sclerostin measurements, and a subgroup of participants with T1D had serum AGEs measurements (Figure 1). A comparison of the clinical characteristics, BMD, and TBS of the participants with or without T1D is presented in Table 1 and Figure 2. Self-reported ethnicity was White in most participants (97.1%). Participants with T1D had diabetes for a mean of 27.6 ± 12.3 yr and a mean HbA_1C_ over the past 3 yr of 7.5 ± 0.9%, 48% had at least one microvascular complication (37% had a retinopathy, 13% a nephropathy, and 22% a neuropathy), and 6 participants had celiac disease. They had a significantly lower femoral neck BMD and TBS than controls. They also had lower serum IGF-1 and phosphate levels, but higher serum-corrected calcium levels. Current smoking and vitamin D supplement intake were more frequent among participants with T1D. The 2 groups did not differ with regard to previous self-reported fractures (43.4% vs 41.5%, *P* = .81) and none of the participants had a history of hip fracture. Six participants with T1D (5.7%) experienced a total of 7 low-trauma fractures (4 ribs, 2 humeri, and 1 wrist fracture).

**Table 1:** Characteristics of participants with and without type 1 diabetes (*n =* 171).

		Type 1 diabetes (*n =* 106)	Controls (*n =* 65)	*P-*value^a^
Demographic and clinical characteristics				
Age (yr)		42.7 ± 14.7	42.2 ± 15.9	.81
Sex (female)		56 (52.8)	35 (53.9)	.90
Postmenopausal (females only)		14 (25.0)	10 (28.6)	.71
Ethnicity (White)		103 (97.2)	63 (96.9)	.99
Body mass index (kg/m^2^)		26.9 ± 5.6	26.1 ± 5.1	.36
Waist circumference (cm)		93.2 ± 14.4	90.4 ± 12.1	.19
Academic degree (university degree)		51 (55.4) (*n =* 92)	54 (83.1) (*n =* 65)	**.0003**
Household income (Canadian dollars)		(*n =* 92)	(*n =* 64)	.52
0–39 999		16 (17.4)	14 (21.9)	
40 000–79 999		22 (23.9)	16 (25.0)	
≥80 000		46 (50.0)	32 (50.0)	
Preferred not to answer		8 (8.7)	2 (3.1)	
Current smoker		8 (7.6)	0 (0.0)	**.02**
Intake of vitamin D supplements		39 (36.8)	14 (21.5)	**.03**
Type 1 diabetes duration (yr)		27.6 ± 12.3		
Mean HbA_1C_ over the past 3 yr (%)		7.52 ± 0.86		
Self-reported hypoglycemic events/wk		3.0 [2.0–5.0]		
Presence of a microvascular complication		51 (48.1)		
Biochemical parameters^b^				
eGFR (mL/min/1.73 m^2^)		104.9 ± 16.4	103.6 ± 14.0	.60
Calcium, corrected for albumin (mmol/L)		2.29 ± 0.08	2.23 ± 0.07	**<.001**
Phosphate (mmol/L)		1.04 ± 0.17	1.12 ± 0.15	**.002**
25OHD (nmol/L)		71.9 ± 28.2	65.4 ± 20.5	.08
PTH (ng/L)		35.0 [25.0–45.0]	37.5 [27.5–49.5]	.41
IGF-1 (μg/L)		114.3 ± 35.5	156.0 ± 52.0	**<.001**
AGEs				
Serum pentosidine (ng/mL) Serum CML (ng/mL)		16.8 [8.2–32.0] (*n =* 61) 48.0 ± 16.8 (*n =* 61)		
Skin AGEs (arbitrary units)^c^		2.15 ± 0.54 (*n =* 101)	1.76 ± 0.35 (*n =* 56)	**<.001**

CML, carboxymethyl lysine; eGFR, estimated glomerular filtration rate; T1D, type 1 diabetes.

Data are expressed as mean ± SD, median [interquartile range], or *n* (percentage). Significant *P*-values (*P* < .05) are shown in bold.

a Unpaired *t*-tests or Mann–Whitney U tests were used to compare means or medians, and χ^2^ tests or Fisher exact tests were used to compare proportions.

b Reference values: eGFR ≥60 mL/min/1.73 m^2^; calcium, corrected for albumin 2.11–2.55 mmol/L; phosphate 0.80–1.45 mmol/L; 25OHD 50–125 nmol/L; PTH 20–100 ng/L; IGF-1 reference values vary according to age: 100–300 μg/L (20–30 yr old), 50–250 μg/L (30–40 yr old) and 35–200 μg/L (>40 yr old).

c Due to the occasional unavailability of the AGE Reader during the study, 101 participants with T1D and 56 controls had skin AGEs measurements.

#### Subgroup of participants with BTMs, sclerostin, and serum AGEs measurements

Among the subgroup of participants who had BTMs and sclerostin measurements, CTX, P1NP, and osteocalcin levels were significantly lower in participants with T1D, while sclerostin levels did not differ between the groups (Figure 2). Supplementary Table S1 compares the clinical characteristics and bone parameters of this subgroup by T1D status. The significant differences in baseline characteristics observed between the participants with T1D and controls who had BTMs and sclerostin measurements were similar to what was observed for the whole study population, except that there was no significant difference in femoral neck BMD, current smoking, and vitamin D supplements intake between the groups. Moreover, BMI, waist circumference, and eGFR were significantly higher in participants with T1D compared to controls. Only one participant from the T1D group had chronic kidney disease (eGFR<60 mL/min/1.73 m^2^). Serum pentosidine and CML were measured only in the subgroup of participants with T1D and were 16.8 [8.2–32.0] ng/mL and 48.0 ± 16.8 ng/mL, respectively. Eleven (18.0%) of the participants with T1D had pentosidine levels higher than the ELISA kit upper limit of detection (50 ng/mL). For these samples, measurements were repeated after dilution to obtain the final values.

### Associations between AGEs and bone parameters

The results from the multiple linear regression analyses of the associations between skin AGEs and BMD at all skeletal sites, TBS, BTMs, and sclerostin are presented in Table 2. As the *P*-values for the interactions between skin AGEs and T1D status were all nonsignificant, participants with T1D and controls were pooled. Higher skin AGEs were significantly associated with lower total hip and femoral neck BMD, TBS, BTMs, and sclerostin in simple linear regression analysis, but were not in the adjusted models. The proportion of the hip BMD, TBS, BTMs, and sclerostin variance that was explained by skin AGEs (*R*-squared value) was approximately 5%–10%, 11%, 5%–13%, and 10%, respectively. The other independent variables included in the models further explained approximately 10%–40% of the bone parameters variance. Moreover, serum pentosidine and CML were not associated with BMD, TBS, BTMs, and sclerostin in simple linear regression analyses. Finally, in the subgroup of participants with T1D and an HbA_1C_ ≥ 7.8%, skin AGEs were not associated with either BMD (*n =* 30), TBS (*n =* 30), BTMs, or sclerostin (*n =* 18). Moreover, serum AGEs were neither associated with BMD (*n =* 20) or TBS (*n =* 20) nor with BTMs or sclerostin (*n =* 20), except for a negative association between serum pentosidine and total hip BMD (*n =* 20) that remained significant after adjusting for age (Supplementary Table S2).

**Table 2:** Associations between skin AGEs and BMD, trabecular bone score, bone turnover markers, and sclerostin in pooled participants with and without type 1 diabetes.

Dependent variable	Independent variables^a^	*ß* coefficient ± SE^b^	*P*-value^b^	Model-adjusted *R*^2^
Lumbar spine BMD (*n* = 156)^c^	Skin AGEs + Age and menopausal status^d^ + BMI + Type 1 diabetes status + eGFR and presence of a microvascular complication	−0.003 ± 0.022 0.040 ± 0.026 0.026 ± 0.025 0.032 ± 0.028 0.022 ± 0.029	.90 .12 .31 .26 .45	−0.0064 0.0510 0.1258 0.1214 0.1189
Total hip BMD (*n* = 156)^c^	Skin AGEs + Age and menopausal status^d^ + BMI + Type 1 diabetes status + eGFR and presence of a microvascular complication	−0.067 ± 0.020 −0.029 ± 0.024 −0.047 ± 0.022 −0.031 ± 0.024 −0.034 ± 0.026	**<.0001** .22 **.04** .20 .19	0.0593 0.1605 0.2779 0.2826 0.2659
Femoral neck BMD (*n* = 156)^c^	Skin AGEs + Age and menopausal status^d^ + BMI + Type 1 diabetes status + eGFR and presence of a microvascular complication	−0.090 ± 0.021 −0.030 ± 0.024 −0.042 ± 0.023 −0.024 ± 0.026 −0.025 ± 0.027	**<.0001** .21 .07 .34 .36	0.0995 0.2118 0.2618 0.2690 0.2529
Distal radius BMD (*n* = 156)^c^	Skin AGEs + Age and menopausal status^d^ + BMI + Type 1 diabetes status + eGFR and presence of a microvascular complication	−0.010 ± 0.012 0.007 ± 0.016 0.005 ± 0.012 0.010 ± 0.013 0.007 ± 0.014	.57 .54 .69 .45 .59	−0.0026 0.4986 0.4979 0.4973 0.4950
TBS (*n* = 156)^c^	Skin AGEs	−0.068 ± 0.016	**<.001**	0.1058
	+ Age and sex	−0.032 ± 0.018	.08	0.1644
	+ Type 1 diabetes status	−0.023 ± 0.020	.25	0.1646
	+ Waist circumference^e^	0.007 ± 0.017	.70	0.4239
	+ eGFR and presence of a microvascular complication	0.006 ± 0.018	.73	0.4203
CTX (*n* = 105)^c^	Skin AGEs	−0.185 ± 0.049	**.0003**	0.1125
	+ Age and menopausal status^d^	−0.164 ± 0.049	**.001**	0.3196
	+ Type 1 diabetes status	−0.028 ± 0.048	.56	0.4944
	+ eGFR	−0.030 ± 0.048	.54	0.4934
P1NP (*n* = 105)^c^	Skin AGEs	−11.78 ± 4.54	**.01**	0.0521
	+ Age and menopausal status^d^	−10.94 ± 4.69	**.02**	0.2299
	+ Type 1 diabetes status	−1.470 ± 4.95	.77	0.3321
	+ eGFR	−1.494 ± 4.98	.76	0.3254
Osteocalcin (*n* = 105)^c^	Skin AGEs	−6.672 ± 1.666	**.0001**	0.1264
	+ Age and menopausal status^d^	−5.819 ± 1.717	**.001**	0.2911
	+ Type 1 diabetes status	−1.868 ± 1.770	.29	0.4153
	+ eGFR	−1.958 ± 1.756	.27	0.4235
Sclerostin (*n* = 105)^c^	Skin AGEs	7.723 ± 2.197	**.0007**	0.0985
	+ Age and menopausal status^d^	−0.074 ± 1.950	.97	0.4573
	+ Type 1 diabetes status	1.246 ± 2.207	.57	0.4605
	+ eGFR	1.030 ± 2.119	.63	0.5031

BTM, bone turnover marker; CML, carboxymethyl lysine; eGFR, estimated glomerular filtration rate; T1D, type 1 diabetes; TBS, trabecular bone score. Significant *P*-values (*P* < .05) are shown in bold.

a Linear regression models were built by adding successively the independent variables in the order they appear in the rows.

b Beta coefficient with SE and *P*-value of the association between skin AGEs and the dependent variable in the linear regression analysis model.

c Interaction between skin AGEs and type 1 diabetes status was not significant in all models (*P*-value for interaction for skin AGEs * T1D status 0.72 for lumbar spine BMD, 0.75 for total hip BMD, 0.58 for femoral neck BMD, 0.75 for distal radius BMD, 0.85 for TBS, 0.82 for CTX, 0.59 for P1NP, 0.97 for osteocalcin and 0.22 for sclerostin). Participants with T1D and controls were pooled in the models.

d Participants were divided into 3 groups for menopausal status: men, premenopausal women, and postmenopausal women

e Waist circumference was preferred over BMI in the model due to its strongest explanation of the TBS variance (higher *R*-squared value) in simple linear regression analysis, and to the multicollinearity between BMI and WC.

### Correlations between skin and serum AGEs as well as between AGEs and mean 3-year HbA_1C_

**Figure 3:**
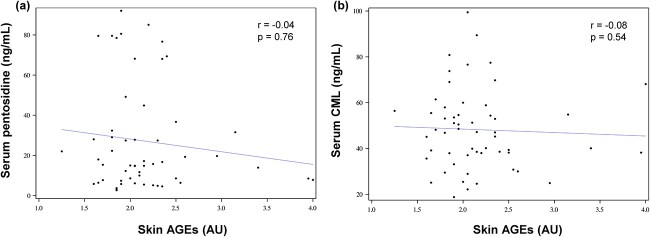
Correlations between skin and serum advanced glycation end products in participants with type 1 diabetes (*n* = 55). Figure Legend: (A) Correlation between skin AGEs and serum pentosidine. (B) Correlation between skin AGEs and serum carboxymethyl-lysine (CML). AU, arbitrary units.

In participants with T1D, there was no correlation between skin AGEs and either serum pentosidine (*r* = −0.04, *P* = .76) or serum CML (*r* = −0.08, *P* = .54) (Figure 3). Moreover, no correlation was observed between serum pentosidine and serum CML (*r* = −0.07, *P* = .61). Finally, mean 3-year HbA_1C_ was not correlated with skin AGEs (*r* = −0.02, *P* = .83), serum pentosidine (*r* = 0.03, *P* = .81), or serum CML (*r* = 0.21, *P* = .12). Partial Spearman correlations adjusted for age and sex yielded similar results.

## Discussion

In this cross-sectional study including participants with or without T1D, we found no significant association between skin AGEs measured by autofluorescence and BMD by DXA, TBS, CTX, P1NP, osteocalcin, and sclerostin. The presence of T1D did not modify these associations. In participants with T1D, we found no associations between serum AGEs (pentosidine and CML) and any of the bone outcomes. Finally, there were no correlations between either skin and serum AGEs or between AGEs and mean 3-year HbA_1C_. However, serum pentosidine was negatively associated with total hip BMD in a sensitivity analysis including participants with an HbA_1C_ ≥ 7.8%. To our knowledge, this is the first study to assess the relationship between both skin and serum AGEs, and BMD, TBS, BTMs, and sclerostin in T1D.

We did not identify any significant association between skin AGEs and BMD by DXA after adjusting for multiple confounders. Our results are in contrast with those of a large cross-sectional study of participants with T1D in which higher skin AGEs were independently associated with a lower total hip, femoral neck, and radius BMD.^15^ Possible explanations for the discrepant results between this study and ours are the smaller sample size, the younger age, and the relatively well-controlled diabetes of our participants. Higher skin AGEs have also been associated with lower BMD in older adults with type 2 diabetes (T2D).^34^^,^^35^ Nevertheless, we identified a significant negative association between serum pentosidine and total hip BMD in a sensitivity analysis including participants with T1D and an HbA_1C_ ≥ 7.8%. The cross-linked fluorescent AGE pentosidine is often the AGE of interest in diabetic bone disease^36^ and, in line with our results, total hip BMD correlated negatively with serum pentosidine levels in a T1D cohort similar to ours (mean age 43 yr; diabetes duration 21 yr; HbA_1C_ 7.7%).^16^ Moreover, higher urinary pentosidine was associated with lower total hip and trochanter BMD and with higher bone loss at the femoral neck and trochanter BMD in older adults with and without T2D.^37^

In line with our findings, published studies did not report a significant association between TBS and AGEs, measured by skin autofluorescence. Findings were consistent in people with T1D or T2D and in those without diabetes.^15^^,^^38-40^ For instance, a large cross-sectional study including over a thousand participants with T1D found no association between skin AGEs measured by skin intrinsic fluorescence with the SCOUT DS spectrometer and TBS.^15^ In this study, long-term diabetes control of the participants was slightly worse than that of our participants (time-weighted mean HbA_1C_ over a 30-year follow-up of 7.9%) and participants were older (mean age of 59 vs 43 yr) and had a longer duration of diabetes (38 vs 28 yr). Skin AGEs measured with the AGE Reader were also not associated with TBS in a large population-based study including participants with and without diabetes (median age 74 yr), but were associated with major osteoporotic fractures and vertebral fractures.^38^ These results suggest that the association between AGEs and fracture risk is not explained by altered TBS.

It has been hypothesized that the absence of association between AGEs and TBS in T1D stems from the differential accumulation of AGEs in trabecular vs cortical bone.^15^ A study in older individuals without diabetes reported that pentosidine content in the trabecular bone is lower than in cortical bone. It was speculated that the faster bone turnover rate of the trabecular bone improves the efficiency of the AGEs removal system.^41^ As TBS assesses trabecular bone architecture, preferential AGEs accumulation in the cortical bone could explain the lack of association between AGEs and TBS. However, more recent in vivo and in vitro studies rather suggest that AGEs accumulate more in the trabecular than in the cortical bone due to the higher surface-to-volume ratio and porous nature of trabecular bone, leading to increased contact between circulating sugars and amino acid residues on collagen.^42-44^ In support of this, trabecular bone from subjects with T1D with a prior fragility fracture exhibited higher pentosidine content than trabecular bone from nondiabetic controls without a history of fracture, but that was not the case for cortical bone.^14^ More recently, postmenopausal women with long-standing T1D exhibited higher pentosidine content in both trabecular and cortical bone, and higher CML in trabecular but not cortical bone, compared with matched controls.^45^ The lower bone turnover rate observed in T1D could decrease AGEs removal in the usually more metabolically active trabecular bone. If AGEs accumulate more in trabecular bone in people with T1D, the nonenzymatic cross-linking process by which AGEs alter bone matrix may not be captured by TBS. Measures that evaluate bone strength and material properties could better unravel the effects of AGEs on bone.^46^^,^^47^

Similar to what we found, a cross-sectional study including only subjects with T1D reported no association between skin AGEs measured with the SCOUT DS spectrometer and serum CTX, tartrate-resistant acid phosphatase 5b (TRACP5b), bone alkaline phosphatase, P1NP, and sclerostin, after adjustment for eGFR.^48^ Noteworthy, study participants had characteristics that are predicted to put them at a higher risk of bone fragility than our participants: they were older (mean age of 59 yr), had a longer diabetes duration (mean of 38 yr), and had a higher HbA_1C_ (mean time-weighted HbA_1C_ over a 30-year follow-up of 8.0%). On the other hand, significant but inconsistent associations have been reported between skin AGEs and BTMs in populations with T2D.^34^^,^^47^ No study assessed the association between serum pentosidine or CML and BTMs or sclerostin in T1D. Although hyperglycemia and AGEs lead to osteocyte dysfunction and induce osteocyte apoptosis in in vivo studies, which could lead to higher levels of sclerostin, no association has been reported between AGEs and sclerostin in humans with diabetes.^49^

Regarding the correlation between skin and blood measurements of AGEs, inconsistent results have been reported. A significant although moderate correlation was reported between skin and total plasma AGEs in people with prediabetes or T2D (*n* = 31, *r* = 0.37, *P* = .04) but not in those with normoglycemia.^50^ Moreover, significant correlations between plasma AGEs measured by high-performance liquid chromatography and skin AGEs have been observed in people with T1D at year 16 of the Epidemiology of Diabetes Interventions and Complications trial.^51^ Nevertheless, another study in people with T1D failed to identify a correlation between skin AGEs and serum levels of CML (*n* = 68, *r* = 0.114, *P* = .37).^52^ More recently, no correlation was identified between skin AGEs and serum CML and pentosidine measured by ELISA in people with moderate to severe chronic kidney disease.^53^ One hypothesis for the lack of correlation between skin and serum AGEs in our study may be that serum AGEs could be acutely influenced by exogenous dietary intake of AGEs.^54^^,^^55^ They could then fluctuate more in the short term than skin AGEs, which tend to represent long-term tissue accumulation. Moreover, the conflicting results could also be explained by the challenge that measuring serum AGEs represents. Indeed, there are concerns about the validity of some commercial immunoassays due to the numerous blood factors that may interfere with immunoassay standardization.^56^

Most studies in adults with T1D—and particularly those with a longer follow-up (≥5–10 yr) and including participants with a mean HbA_1C_ ≥ 7.8%—identified a positive correlation between cumulative HbA_1C_ and skin^57-59^ or plasma AGEs.^51^^,^^60^ However, some studies in children and adults with T1D did not report a significant correlation between cumulative HbA_1C_ < 5 yr or HbA_1C_ at one timepoint and skin^58^^,^^61^ or serum AGEs.^52^ The lack of correlation between mean 3-year HbA_1C_ and AGEs in our study could be explained by (1) the relatively short-term cumulative HbA_1C_ and well-controlled T1D and (2) the fact that AGEs accumulation is the result of various mechanisms in addition to hyperglycemia, such as oxidative stress and decreased renal clearance of AGEs precursors.^62^

A strength of our study is the measurement of both skin and serum AGEs as well as fluorescent (pentosidine) and nonfluorescent (CML) serum AGEs to better capture the effects of AGEs on bone fragility in T1D. Nevertheless, our study has limitations. First, our population may appear at a lower risk of bone fragility than previously reported cohorts of subjects with and without diabetes (younger age, well-controlled diabetes, no history of fragility fracture, higher education level), which may explain the lack of significant associations. Moreover, the relatively small number of participants with T1D precluded the conduct of additional subgroup analyses. Second, we based our analyses on a single fasting serum pentosidine and CML measurement, which might not accurately reflect nonfasting and long-term pentosidine and CML levels. Third, we did not measure soluble receptors of AGEs (sRAGE), including endogenous secretory receptors of AGEs (esRAGE). As serum sRAGE-to-pentosidine and esRAGE-to-pentosidine ratios have been more strongly correlated to fracture risk than serum pentosidine alone in T2D^63^ and in older men,^64^ respectively, they could represent a better mean to assess the bone fragility related to AGEs in diabetes. Fourth, the application of lotions or creams on the forearm the days prior to the study visit—which can falsely elevate skin AGEs measurements^17^—was not consistently collected. Moreover, use of more precise imaging techniques such as high-resolution peripheral quantitative computed tomography (HR-pQCT) or pQCT or bone histomorphometry could have captured a differential effect of AGEs on trabecular and cortical bone. Finally, as most of our participants self-reported as White, results may not be generalizable to other ethnic groups.

## Conclusion

In conclusion, we did not identify significant associations between either skin or serum AGEs and BMD, TBS, BTMs, and sclerostin in people with relatively well-controlled T1D and people without diabetes. However, as some studies reported an association between serum AGEs and fractures in people with T1D, further research is needed and could include participants with more deteriorated diabetes control, assess the relationship between the AGE-RAGE axis and bone outcomes, and use more sophisticated techniques to evaluate bone outcomes such as HR-pQCT and bone histomorphometry.

## Supplementary Material

Supplementary_Material_FINAL_revised_clean_ziad018

## Data Availability

The data that support the findings of this study are available from the corresponding author upon reasonable request.

## Permission to reproduce material from other sources

No material was reproduced from other sources.

## Clinical trial registration number

NCT04064437 (ClinicalTrials.gov Identifier).
