# Supplementary material for: Advanced glycation end products are not associated with bone mineral density, trabecular bone score, and bone turnover markers in adults with and without type 1 diabetes: a cross-sectional study
Source: JBMR Plus. 2024 Jan 4;8(3):ziad018. doi: 10.1093/jbmrpl/ziad018 (PMC10945729; doi:10.1093/jbmrpl/ziad018)
Supplement: Supplementary_Material_FINAL_revised_clean_ziad018 [file supplementary_material_final_revised_clean_ziad018.docx]

**Supplementary Material**

**Table S1. Characteristics of Participants with Bone Turnover Markers, Sclerostin, and Serum Advanced Glycation End Products Measurements (n=118).**

|  | **Type 1 Diabetes (n=61)** | **Controls (n=57)** | ***P* value**^a^ |
| --- | --- | --- | --- |
| Demographic and clinical characteristics |  |  |  |
| Age (years) | 42.1 ± 12.6 | 42.8 ± 16.6 | 0.80 |
| Sex (female) | 30 (49.2) | 28 (49.1) | 0.99 |
| Postmenopausal (females only) | 4 (13.3) | 9 (15.8) | 0.09 |
| Body mass index (kg/m^2^) | 27.8 ± 6.2 | 25.7 ± 4.6 | **0.04** |
| Waist circumference (cm) | 95.2 ± 14.9 | 90.1 ± 11.6 | **0.04** |
| Current smoker | 3 (4.9) | 0 (0.0) | 0.24 |
| Intake of vitamin D supplements | 21 (34.4) | 13 (22.8) | 0.16 |
| Type 1 diabetes duration (years) | 27.2 ± 9.7 | - | - |
| Mean HbA_1C_ over the past 3 years (%) | 7.52 ± 0.74 | - | - |
| Self-reported hypoglycemic events/week | 3.0 [2.0-5.0] | - | - |
| Presence of a microvascular complication | 30 (49.2) | - | - |
| Biochemical parameters^b^ |  |  |  |
| eGFR (mL/min/1.73 m^2^) | 107.6 ± 13.4 | 102.1 ± 14.0 | **0.03** |
| Calcium, corrected for albumin (mmol/L) | 2.29 ± 0.09 | 2.23 ± 0.07 | **0.0003** |
| Phosphate (mmol/L) | 1.03 ± 0.16 | 1.13 ± 0.14 | **0.0006** |
| 25-hydroxyvitamin D (nmol/L) | 71.3 ± 28.9 | 66.5 ± 20.5 | 0.30 |
| PTH (ng/L) | 36.0 [28.0-51.0] | 37.0 [26.5-47.5] | 0.94 |
| IGF-1 (ug/L) | 111.0 ± 34.8 | 153.5 ± 49.9 | **<0.001** |
| AGEs  Serum pentosidine (ng/mL)  Serum CML (ng/mL)  Skin AGEs (arbitrary units)^c^ | 16.8 [8.2-32.0]  48.0 ± 16.8  2.15 ± 0.52 (n=56) | -  -  1.75 ± 0.36 (n=49) | -  -  **<0.001** |
| Bone mineral density |  |  |  |
| Lumbar spine (g/cm^2^)  T-score  Z-score | 1.020 ± 0.114  -0.45 ± 1.06  -0.02 ± 1.86 | 1.013 ± 0.153  -0.54 ± 1.32  -0.10 ± 1.22 | 0.78  0.68  0.77 |
| Total hip (g/cm^2^)  T-score  Z-score | 0.964 ± 0.122  -0.22 ± 0.97  0.08 ± 0.91 | 0.985 ± 0.151  -0,03 ± 1.07  0.29 ± 0.94 | 0.40  0.30  0.23 |
| Femoral neck (g/cm^2^)  T-score  Z-score | 0.828 ± 0.130  -0.48 ± 1.09  -0.003 ± 1.02 | 0.853 ± 0.149  -0.30 ± 1.22  0.24 ± 1.01 | 0.33  0.41  0.21 |
| Distal radius (g/cm^2^)  T-score  Z-score | 0.750 ± 0.090  -0.12 ± 1.15  0.31 ± 1.13 | 0.749 ± 0.089  -0.12 ± 1.14  0.39 ± 1.06 | 0.94  0.99  0.70 |
| Trabecular bone score (unitless)  T-score  Z-score | 1.423 ± 0.115  -0.41 ± 1.12  0.28 ± 1.18 | 1.465 ± 0.089  0.004 ± 0.81  0.71 ± 0.70 | **0.03**  **0.02**  **0.02** |
| Bone turnover markers and sclerostin  CTX (ng/mL)  P1NP (ng/mL)  Osteocalcin (ng/mL)  Sclerostin (pmol/L) | 0.297 ± 0.147  40.6 ± 16.1  16.6 ± 5.7  30.2 ± 12.5 | 0.564 ± 0.273  59.5 ± 25.0  24.9 ± 9.2  32.9 ± 10.8 | **<0.001**  **<0.001**  **<0.001**  0.21 |

AGEs, advanced glycation end products; CML, carboxymethyl lysine; CTX, C-terminal crossed-linked telopeptide of type 1 collagen; eGFR, estimated glomerular filtration rate; P1NP, procollagen type 1 N-terminal propeptide.

Data are expressed as mean ± SD, median [interquartile range], or n (percentage).

^a^Unpaired t-tests or Mann-Whitney U tests were used to compare means or medians, and χ^2^ tests or Fisher exact tests were used to compare proportions. ^b^Reference values: eGFR ≥ 60 mL/min/1.73 m^2^; calcium, corrected for albumin 2.11-2.55 mmol/L; phosphate 0.80-1.45 mmol/L; 25-hydroxyvitamin D 50-125 nmol/L; PTH 20-100 ng/L; IGF-1 reference values vary according to age: 100-300 ug/L (20-30 years old), 50-250 ug/L (30-40 years old) and 35-200 ug/L (>40 years old). ^c^Due to the occasional unavailability of the AGE Reader during the study, 56 participants with T1D and 49 controls had skin AGEs measurements.

**Table S2. Associations between Skin and Serum Advanced Glycation End Products and Bone Mineral Density, Trabecular Bone Score, Bone Turnover Markers, and Sclerostin in the Subgroup of Participants With HbA_1C_ ≥7.8%.**

| **Dependent variable** | **Independent variable** | ***ß* coefficient ± SE^a^** | ***P* value^a^** |
| --- | --- | --- | --- |
| Lumbar spine BMD | Skin AGEs (n=30) | 0.0006 ± 0.049 | 0.99 |
|  | Serum CML (n=20) | -0.002 ± 0.002 | 0.35 |
|  | Serum pentosidine (n=20) | -0.0001 ± 0.0008 | 0.87 |
| Total hip BMD | Skin AGEs (n=30) | -0.014 ± 0.044 | 0.75 |
|  | Serum CML (n=20) | 0.0006 ± 0.002 | 0.77 |
|  | Serum pentosidine (n=20) | -0.002 ± 0.0008 | **0.02^b^** |
| Femoral neck BMD | Skin AGEs (n=30) | -0.059 ± 0.048 | 0.23 |
|  | Serum CML (n=20) | 0.002 ± 0.002 | 0.31 |
|  | Serum pentosidine (n=20) | -0.001 ± 0.0009 | 0.21 |
| Distal radius BMD | Skin AGEs (n=30) | 0.031 ± 0.030 | 0.32 |
|  | Serum CML (n=20) | -0.002 ± 0.001 | 0.20 |
|  | Serum pentosidine (n=20) | -0.0002 ± 0.0007 | 0.75 |
| TBS | Skin AGEs (n=30) | -0.027 ± 0.049 | 0.59 |
|  | Serum CML (n=20) | -0.002 ± 0.002 | 0.31 |
|  | Serum pentosidine (n=20) | -0.0005 ± 0.001 | 0.67 |
| CTX | Skin AGEs (n=18) | -0.026 ± 0.054 | 0.64 |
|  | Serum CML (n=20) | -0.005 ± 0.003 | 0.11 |
|  | Serum pentosidine (n=20) | 0.0003 ± 0.002 | 0.85 |
| P1NP | Skin AGEs (n=18) | 2.572 ± 6.655 | 0.70 |
|  | Serum CML (n=20) | -0.218 ± 0.318 | 0.50 |
|  | Serum pentosidine (n=20) | -0.115 ± 0.153 | 0.46 |
| Osteocalcin | Skin AGEs (n=18) | -0.445 ± 3.102 | 0.89 |
|  | Serum CML (n=20) | -0.049 ± 0.131 | 0.71 |
|  | Serum pentosidine (n=20) | -0.027 ± 0.063 | 0.68 |
| Sclerostin | Skin AGEs (n=18) | 6.828 ± 5.596 | 0.24 |
|  | Serum CML (n=20) | -0.302 ± 0.184 | 0.12 |
|  | Serum pentosidine (n=20) | 0.002 ± 0.095 | 0.99 |

AGEs, advanced glycation end products; BMD, bone mineral density; CML, carboxymethyl lysine; CTX, C-terminal crossed-linked telopeptide of type 1 collagen; P1NP, procollagen type 1 N-terminal propeptide; TBS, trabecular bone score.

^a^Beta coefficient with standard error and p value of the association between the independent AGEs variable and the dependent bone parameter variable in simple linear regression analysis.^b^The association remained significant after adjusting for age (beta coefficient= -0.002 ± 0.0008, p=0.02, model adjusted R^2^=0.2197).
